# Characterization of *Mycobacterium bovis* from Humans and Cattle in Namwala District, Zambia

**DOI:** 10.1155/2014/187842

**Published:** 2014-04-14

**Authors:** Sydney Malama, Tone Bjordal Johansen, John Bwalya Muma, Musso Munyeme, Grace Mbulo, Adrian Muwonge, Berit Djønne, Jacques Godfroid

**Affiliations:** ^1^Institute of Economic and Social Research, University of Zambia, P.O. Box 30900, Lusaka, Zambia; ^2^Norwegian Veterinary Institute, P.O. Box 750, 0106 Oslo, Norway; ^3^Department of Disease Control, School of Veterinary Medicine, University of Zambia, P.O. Box 32379, Lusaka, Zambia; ^4^Tuberculosis Laboratory, Department of Microbiology and Pathology, University Teaching Hospital, P/Bag RW1X, Lusaka, Zambia; ^5^The Roslin Institute, College of Medicine and Veterinary Medicine, University of Edinburgh, Easter Bush, Roslin, Midlothian EH25 9RG, UK; ^6^Department of Arctic and Marine Biology, University of Tromsø-The Arctic University of Norway, Stakkevollveien 23, 9010 Tromsø, Norway

## Abstract

Tuberculosis remains a major public health problem in Zambia. While human to human transmission of * Mycobacterium tuberculosis * is of major importance in driving the tuberculosis epidemic, the impact of * Mycobacterium bovis * transmission from infected cattle is largely unknown. This cross-sectional study aimed at molecular characterization of * M. bovis * in humans and cattle. A total of 100 human sputum samples and 67 bovine tissues were collected and analyzed for the presence of mycobacteria. Of 65 human samples that harbored acid fast bacteria (AFB), 55 isolates were obtained of which 34 were identified as * M. tuberculosis * and 2 as * M. bovis*. AFB-positive bovine samples (*n* = 67) yielded 47 mycobacterial isolates among which 25 were identified as * M. bovis *and no * M. tuberculosis *was found. Among the * M. bovis * isolates, spoligotyping revealed a high homogeneity in genotypes circulating in Namwala district. Human and cattle isolates shared identical MIRU-VNTR genotypes, suggesting that transmission between the two hosts may occur. Therefore, this study has documented zoonotic TB in human patients in Namwala district of Zambia. However, further molecular epidemiological studies in the study area are recommended.

## 1. Introduction

*Mycobacterium tuberculosis *is the most common cause of tuberculosis (TB) in humans but an unknown proportion of cases are due to* Mycobacterium bovis*.* M. bovis* is mainly responsible for bovine tuberculosis (bTB) in cattle and in a wide range of both domestic and wild animals. It is also a known cause of zoonotic tuberculosis in humans, which can appear indistinguishable with regard to pathogenesis, lesions, and clinical findings to that caused by* M. tuberculosis* [1, 2].* M. bovis* shows a high degree of virulence for both humans and animals [1].

The WHO reported in 1998 that 3.1% of TB in humans worldwide is attributable to* M. bovis* and that 0.4–10% of sputum isolates from patients in African countries could be* M. bovis *[3]. Zoonotic TB is now acquiring increasing recognition in developing countries, including Zambia, as animals and humans share the same environment [4]. This has also prompted researchers to evaluate its impact on human health, particularly among the pastoral communities. In Uganda, Oloya et al. (2008) found a prevalence rate of 7%* M. bovis *in humans suffering from cervical lymphadenitis in a pastoral community in the Karamoja region [5], while a study conducted in Tanzania in 2001 on human patients with cases of lymphadenitis reported that 16% of the cases were due to* M. bovis *[6]. In Zaire (now Democratic Republic of Congo),* M. bovis* was isolated from gastric secretions in two of five patients with pulmonary TB [7]. Isolation of* M. bovis* from sputum samples of patients with pulmonary TB has also been reported from Nigeria in which 3.9% of* M. tuberculosis* complex (MTC) isolates were* M. bovis *[8]. In a more recent study, Gumi et al. (2012) found a link in the transmission of TB between livestock and pastoralists of South East Ethiopia [9]. Zoonotic TB is therefore a public health threat in developing countries [10]. It is, however, important to differentiate* M. tuberculosis* from* M. bovis* to warrant good patient management with regard to treatment regimen [11, 12].

The tracing of routes of transmission of TB is equally important in the control of the disease. Therefore, the need for molecular techniques permitting molecular typing and differentiation of isolates is evident. These tools can help to determine the origin of outbreaks, show the relationships of TB in domestic and wild animals, and identify the sources of human infection [13]. Bovine TB has been reported to be endemic in traditional livestock and in wildlife in Namwala district [14–16]. It has, however, been speculated that zoonotic TB could occur in humans based on the cultural activities of the people in this area such as consumption of raw milk [17, 18]. This study aimed at molecular identification of* M. bovis* in humans and cattle as well as documentation of its zoonotic significance in Namwala district of Zambia.

## 2. Material and Methods 

### 2.1. Ethics Statement

The study was approved by Institutional Review Board (ERES converge IRB) ethical review committee (ref. number: 2012-Mar-001) and permission to perform the study in the district was obtained from the District Medical Officer (DMO) and the Provincial Veterinary Officer through the District Veterinary Officer. All human study subjects provided informed written consent either in English or the local language.

### 2.2. Study Site

Namwala district is situated in the southern province of Zambia. It covers an estimated total area of about 10,000 square kilometers and lies between latitudes 15 and 17° S of the equator and longitude 25 and 27° E. The greater expanse of its traditional land is covered by the Kafue flood plains, which offer nutritive varieties of rich lush green grass for both cattle and wildlife for a greater part of the year than the surrounding Savannah woodlands, and support close to 300,000 cattle [19] and about 102,000 humans [20]. Agriculture is the main economic activity, and majority of the people keep livestock. Humans and animals commonly share the same microenvironments and water points, especially in dry seasons and during years of droughts [18]. Sputum samples were collected from humans at three satellite health facilities: the Central Namwala District Hospital, the Maala Rural Health Centre, and the Chitoongo Rural Health Centre. Sampling from cattle carcasses was performed at one of the two districts commercial abattoirs.

### 2.3. Study Design

The study was designed as a cross section study. Suspected TB patients seeking medical attention at the Central Namwala District Hospital, the Maala Rural Health Centre, and the Chitoongo Rural Health Centre comprised the target population for the human study. For the cattle study, the target population comprised cattle carcasses showing gross tuberculous-like lesions at meat inspection at one of the two abattoirs in the Namwala district. Our sampling therefore was purposive, where subjects are selected based on the above described characteristics in order to increase the chances of isolating* M. bovis*. The cattle that were slaughtered at the abattoir were drawn from the villages where patients seeking medical attention at the three health facilities came from.

### 2.4. Human Samples

From April 2011 to July 2012 a total of 150 suspected pulmonary TB patients seeking medical attention at the three sampling sites were screened. The suspected pulmonary TB patients were patients who had a cough of more than two weeks; they also reported loss of appetite and night sweats. However, only 110 patients submitted the sputum samples. Ten sputum samples were discarded due to poor quality leaving 100 samples for further processing. After routine microscopy by the Ziehl-Neelsen staining method, samples containing acid fast bacilli (AFB) were stored in cetylpyridinium chloride (Difco, Detroit, MI) transport medium and kept at ambient temperature at Namwala District Hospital. All samples were then taken to the Chest Diseases Laboratory where they were decontaminated and cultured on duplicate Lowenstein-Jensen media (one containing glycerol and the other pyruvate) (BD BBL; Franklin Lakes, NJ, USA) as described by Vestal and Kubica [21]. Culture tubes were incubated at 37°C and read weekly for growth for at least eight weeks. Successfully grown cultures were transported to University of Zambia, School of Veterinary Medicine, for storage and onward shipment of isolates to the Norwegian Veterinary Institute in Oslo for further analysis.

### 2.5. Cattle Samples

From April 2011 to June 2012, a total of 288 slaughtered cattle at one of the two abattoirs were examined, of which 67 had lesions compatible with bTB and were sampled. It should be noted here that the animals from which samples were obtained were being processed as part of the routine work of the abattoir. A set of tissues from each carcass were homogenized with sterile sand and distilled water then decontaminated with 4% NaOH for 30 min [22] and centrifuged at 3500 rpm (1068 ×g) for 30 min. The sediments of these decontaminated homogenates were inoculated in duplicate, one on Stonebrink with pyruvate and the other on Middlebrook 7H10 (BD Diagnostics, MD) slants, and incubated aerobically at 37°C for 8 weeks with weekly observation. Isolates were identified as* M. tuberculosis* complex by staining for acid alcohol-fastness.

### 2.6. DNA Extraction and Purification

Mycobacterial colonies grown on solid media were harvested as a loop full of colony material, suspended in 200 *μ*L of molecular grade water, and heated at 95°C for 20 minutes. The heat killed cells were then cooled and the DNA was purified using the NucliSENS easyMAG automated machine, according to the manufacturer's protocol (Biomerieux, The Netherlands). The DNA was stored at −20°C for further analysis.

### 2.7. Identification of Mycobacteria

All isolates were examined by IS6110 real time PCR as described by Agdestein et al. [23]. IS6110 is considered specific for the* M. tuberculosis* complex (MTC) and the real time PCR was used as a screening test to identify isolates belonging to the MTC [24]. The standard multiplex PCR amplification of the genomic region of difference was performed as described [25] at the Norwegian Veterinary Institute. A set of primers targeting the RD1, RD4, RD9, and RD12 regions were used. This generated a deletion profile that allowed speciation of the isolate.

### 2.8. Spoligotyping and MIRU-VNTR

Spoligotyping was performed as previously described by Kamerbeek et al. [26] at the University of Zambia, repeated at the Norwegian Veterinary Institute, and confirmed at Genoscreen in Lille, France. Spoligotyping and MIRU-VNTR were done only on* M. bovis* isolates from humans and cattle. The variable-number tandem repeat (VNTR) method based on nine targeted loci (ETR-A, ETR-B, ETR-D, QUB 11a, QUB 11b, QUB 3232, QUB 26, MIRU 26, and MIRU 31) as recommended by the European reference Laboratory (EURL lab) for* M. bovis *was used. The analysis was carried out as described [27], and primers previously reported were used to amplify the ETR-A and ETR-B loci [28], VNTR 580 and VNTR 2461 loci [29], and QUB 3232, QUB 11a, QUB 11b, and QUB 26 loci [27]. To detect differences in repeat numbers, the size of the PCR products in base pairs was determined using the Agilent Bioanalyzer (Agilent Technologies, CA, USA).

### 2.9. Data Assembly and Analysis

Spoligotyping data in binary form from Genoscreen and the MIRU-VNTR data generated at the Norwegian Veterinary Institute were entered and validated in Excel^©^2007. These were then copied into the SpolDB4.0 [30] database to establish the lineage, sublineage, and spoligotype international types (SIT) designations and also in Bionumerics 6.1 soft for cluster analysis using the unrooted UPGMA tree.

The allelic diversity (*h*) for each locus was calculated using the method described by Selander et al. [31]. The formula *h* = 1 − Σ*xi*²[*n*/(*n* − 1)], where *x* is the frequency of the *i*th allele at the locus, *n* is the number of isolates in the analysis, and *n*/(*n* − 1) is the correction factor for bias in small samples. The discriminatory power (DI) was computed using the Hunter-Gaston discrimination index [32].

## 3. Results 

### 3.1. Identification

In humans, out of the 100 sputum samples, 65 were found to contain AFB. Mycobacteria were detected in 55 samples, and based on IS6110 real time PCR and deletion analysis, 36 isolates belonged to the MTC. Two isolates were identified as* M. bovis *and 34 were* M. tuberculosis*.

In cattle carcasses, AFB was detected in 67 tissue samples, while live mycobacteria were detected by culture from 47 samples. Based on IS6110 real time PCR and deletion analysis, 25 isolates belonged to MTC and were identified as* M. bovis*.

### 3.2. Molecular Characterization Based on Spoligotyping and MIRU-VNTR

**Figure 1:**
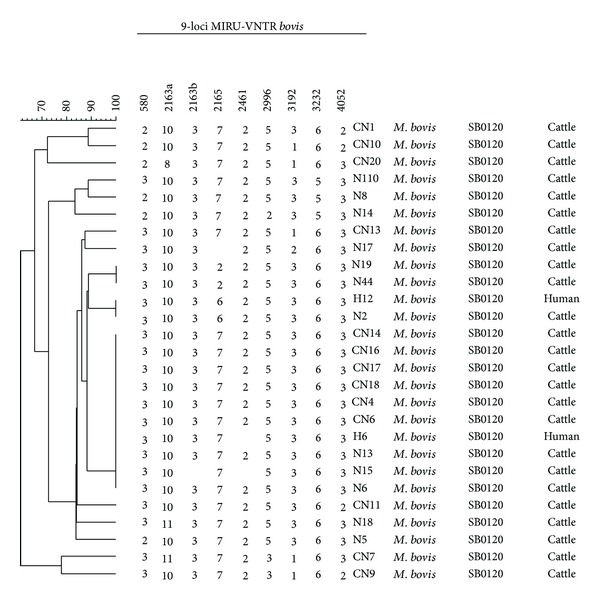
Clustering of representative* M. bovis* isolates from Namwala district in Zambia based on 9-loci MIRU-VNTR. The dendrogram was generated using Bionumerics 6.1 version software.

The total of 27* M. bovis *isolates was characterized by spoligotyping, 25 originating from cattle and 2 from humans. They all showed identical profiles characterized by absence of spacers 3, 9, 16, and 39–43, SB 0120/SIT 482. Based on the 9-loci MIRU-VNTR, the 27 isolates were differentiated into sixteen different patterns. One human isolate (H12) had identical VNTR pattern with one cattle isolate (N2), while the other human isolate belonged to the same cluster as nine other isolates from cattle but never amplified at one locus (2461) (Figure 1). There were only three clusters observed. The largest cluster had ten isolates while the other two had two each. The other thirteen were singletons (Figure 1). Locus 2461 was not amplified in one isolate and showed no discrimination while locus 2163b was not amplified in one isolate and showed no discrimination. One locus 3232 showed no discrimination in all the isolates. Locus 2165 was not amplified in one isolate but discriminated the isolates fairly. The other six loci, however, were amplified in all isolates and showed good discrimination.

## 4. Discussion

This study reports the isolation of mycobacteria from cattle and humans and also utilization of spoligotyping and 9-loci MIR-VNTR typing to study the diversity and relatedness of* M. bovis* isolates from humans and cattle in Namwala district. We observed that infections by* M. bovis* and* M. tuberculosis* were prevalent in cattle and humans, respectively. This is generally in agreement with what has been observed regarding the host preference of these two members of MTC [33]. However, two isolates of* M. bovis* were recovered from human sputum, suggesting human exposure to zoonotic TB. Based on the spoligotyping results, the SB 0120/SIT 482, lacking spacers 3, 9, 16, and 39−43 in the hybridization pattern, was the circulating strain revealed in this area. This indicates that highly homogenous isolates of* M. bovis* are circulating in both cattle and humans in the district. The same spoligotype has been isolated from cattle in Algeria, Brazil, France, South Africa, and Zambia [16, 34–37] and from humans in Italy and Germany [38–40]. The isolated spoligotype SB 0120 belongs to the BCG family but has not undergone chromosomal deletion (RDAf1) as the one observed in some parts of Africa, which is identified by the absence of spacer 30 in the standard spoligotyping scheme [41]. It is also different from the other observed in East Africa which has undergone a chromosomal deletion of RDAf2 and identified by the absence of spacers 3 to 7 [42]. However, combining spoligotyping with MIRU-VNTR results, it has been established that the observed profiles could be unique to Zambia. They are different from those observed in other southern African countries such as South Africa and elsewhere [43, 44].

Based on the 9-loci MIRU-VNTR, isolates were discriminated into 16 different MIRU-VNTR profiles with three different clusters. The largest cluster had 10 isolates, followed by two which had two isolates. The remaining isolates were singletons. Six loci (0580, 2163a, 2996, 3192, 3232, and 4052) did amplify in all the isolates and gave significant allelic diversity index making them suitable for discrimination of* M. bovis* in such a setup. Therefore, these loci discriminated the isolates relatively well and corroborate with what was found in studies done in South Africa and Spain [36, 44, 45]. Despite the close contact between humans and livestock in the study area, including consumption of raw milk by the local people, the observed* M. bovis *infection in people was lower than what has been observed in other studies [6]. This could be attributed to the type of sample utilized in the study. Sputum was utilized as opposed to lymph node biopsy.* M. bovis* is believed to be highly associated with extra pulmonary TB [1, 6, 46]. In a study done in Uganda in which lymph node biopsies were sampled, a high incidence of* M. bovis* was reported [5]. However, the present study shows that* M. bovis* is also responsible for pulmonary TB infections in humans and is in agreement with what was observed elsewhere [3]. It was observed in this study that, in Namwala district, human and cattle share similar strains of* M. bovis*. This could be seen in the identical spoligotype and MIRU-VNTR patterns in one isolate from a patient and one isolate from cattle. As mentioned, the likely human exposure in this context is consumption of raw or soured cattle milk. This suggests that* M. bovis* transmission between the species occurs. The sharing of common* M. bovis* strains between human and cattle observed in this study corroborates with what was observed in a recent study done in pastoral region in Ethiopia [9]. The clustering of the human isolates with cattle isolates could mean that related isolates of* M. bovis* are circulating in the cattle and humans in the study area.

The present study has also shown that MIRU-VNTR based on nine loci is a better tool than spoligotyping for typing of* M. bovis* isolates; however, the loci showing no discrimination between isolates as observed in this study should be omitted in order to achieve a higher discriminatory power. It has been observed elsewhere that MIRU-VNTR is an appropriate typing tool for* M. bovis* [47, 48]. The use of 9 loci, instead of 24, was employed following recommendations by the European Reference Laboratory (EURL) and the method has shown to be a suitable alternative in resource strained countries since it utilises few loci.

In conclusion, this study has documented zoonotic TB in human patients in Namwala district of Zambia. Isolation of* M*.* bovis* from human sputum suggests potential of human to human infection, especially in closed populations such as prisons. This study further demonstrates that zoonotic TB is of public health significance in pastoral communities of Africa that demands interventions. Since* M*.* bovis* infects both human and animal hosts, its control demands an intersectoral approach between the medical and veterinary professionals. With the growing global realization that pathogens do not respect traditional epistemological divides, the “One Health” initiative has emerged to advocate for closer collaboration across the health disciplines and in this particular case is being advocated for in the control of TB infections in this area. The study has further shown that MIRU-VNTR with these nine loci is a well-suited tool for* M*.* bovis* typing in resource poor settings, as the case may be in many African countries. However, further molecular epidemiological studies with more discriminatory loci, a larger sample size, and lymph node biopsies from human subjects are recommended. Our study has reemphasized that the choice of loci being recommended for the typing of* M*.* bovis* in Europe may not be optimal in Africa and Namwala district in particular.
